# Generative Large Language Models in Electronic Health Records for Patient Care Since 2023: A Systematic Review

**DOI:** 10.1101/2024.08.11.24311828

**Published:** 2024-08-19

**Authors:** Xinsong Du, Zhengyang Zhou, Yifei Wang, Ya-Wen Chuang, Richard Yang, Wenyu Zhang, Xinyi Wang, Rui Zhang, Pengyu Hong, David W. Bates, Li Zhou

**Affiliations:** 1 Division of General Internal Medicine and Primary Care, Brigham and Women’s Hospital, Boston, Massachusetts 02115; 2 Department of Medicine, Harvard Medical School, Boston, Massachusetts 02115; 3 Department of Biomedical Informatics, Harvard Medical School, Boston, Massachusetts 02115; 4 Department of Computer Science, Brandeis University, Waltham, MA 02453; 5 Division of Nephrology, Department of Internal Medicine, Taichung Veterans General Hospital, Taichung, Taiwan, 407219; 6 Department of Post-Baccalaureate Medicine, College of Medicine, National Chung Hsing University, Taichung, Taiwan, 402202; 7 School of Medicine, College of Medicine, China Medical University, Taichung, Taiwan, 404328; 8 Division of Computational Health Sciences, University of Minnesota, Minneapolis, MN 55455; 9 Department of Health Policy and Management, Harvard T.H. Chan School of Public Health, Boston, MA 02115

## Abstract

**Background:**

Generative Large language models (LLMs) represent a significant advancement in natural language processing, achieving state-of-the-art performance across various tasks. However, their application in clinical settings using real electronic health records (EHRs) is still rare and presents numerous challenges.

**Objective:**

This study aims to systematically review the use of generative LLMs, and the effectiveness of relevant techniques in patient care-related topics involving EHRs, summarize the challenges faced, and suggest future directions.

**Methods:**

A Boolean search for peer-reviewed articles was conducted on May 19^th^, 2024 using PubMed and Web of Science to include research articles published since 2023, which was one month after the release of ChatGPT. The search results were deduplicated. Multiple reviewers, including biomedical informaticians, computer scientists, and a physician, screened the publications for eligibility and conducted data extraction. Only studies utilizing generative LLMs to analyze real EHR data were included. We summarized the use of prompt engineering, fine-tuning, multimodal EHR data, and evaluation matrices. Additionally, we identified current challenges in applying LLMs in clinical settings as reported by the included studies and proposed future directions.

**Results:**

The initial search identified 6,328 unique studies, with 76 studies included after eligibility screening. Of these, 67 studies (88.2%) employed zero-shot prompting, five of them reported 100% accuracy on five specific clinical tasks. Nine studies used advanced prompting strategies; four tested these strategies experimentally, finding that prompt engineering improved performance, with one study noting a non-linear relationship between the number of examples in a prompt and performance improvement. Eight studies explored fine-tuning generative LLMs, all reported performance improvements on specific tasks, but three of them noted potential performance degradation after fine-tuning on certain tasks. Only two studies utilized multimodal data, which improved LLM-based decision-making and enabled accurate rare disease diagnosis and prognosis. The studies employed 55 different evaluation metrics for 22 purposes, such as correctness, completeness, and conciseness. Two studies investigated LLM bias, with one detecting no bias and the other finding that male patients received more appropriate clinical decision-making suggestions. Six studies identified hallucinations, such as fabricating patient names in structured thyroid ultrasound reports. Additional challenges included but were not limited to the impersonal tone of LLM consultations, which made patients uncomfortable, and the difficulty patients had in understanding LLM responses.

**Conclusion:**

Our review indicates that few studies have employed advanced computational techniques to enhance LLM performance. The diverse evaluation metrics used highlight the need for standardization. LLMs currently cannot replace physicians due to challenges such as bias, hallucinations, and impersonal responses.

## 1. Introduction

The Transformer architecture, introduced by Vaswani et al. in 2017, marked a significant breakthrough in natural language processing (NLP) by enabling models to handle vast amounts of textual data with unparalleled efficiency and effectiveness.^1^ This architecture relies on self-attention mechanisms to process input sequences in parallel, allowing it to capture long-range dependencies and contextual relationships more effectively than previous models. Building on this foundation, two major categories of language models (LLMs) have emerged: encoder-based models and generative models.

Encoder-based models, such as BERT (Bidirectional Encoder Representations from Transformers),^2^ Longformer,^3^ NYUTron,^4^ GatorTron,^5^ focus on understanding and encoding the input text into dense representations that capture the nuanced meanings and relationships within the data. These models excel in tasks like text classification and named entity recognition where deep contextual understanding is crucial.

In contrast, generative models, such as GPT (Generative Pre-trained Transformer),^2^ primarily leverage the Transformer’s decoder architecture to comprehend and generate human-like text. These models are designed to produce coherent and contextually appropriate language, making them highly effective for applications like content creation, dialogue systems, and even complex problem-solving tasks. The generative capabilities of these models open new possibilities for human-machine interaction, pushing the boundaries of what AI can achieve in language-based tasks.

With the release of ChatGPT^6^ on November 30^th^, 2022, recent advancements in transformer-based generative large language models (LLMs) have significantly transformed the landscape of natural language processing (NLP) and artificial intelligence (AI)^1,2,7^. These models, distinguished by their substantial size and intricate architecture, have gained widespread recognition in both academic and industrial domains due to their extraordinary capability to comprehend and generate human-like reasoning^8^. With billions to trillions of parameters, they are exceptionally proficient in capturing complex linguistic patterns and subtleties, achieving unprecedented levels of accuracy and depth.

Given the remarkable capabilities of generative LLMs in processing text data, there has been a surge in research exploring their applications in healthcare. Numerous studies have reviewed and synthesized recent advancements in applying LLMs to various healthcare domains.^9–12^ Some researchers have evaluated LLMs’ ability to answer healthcare-related questions by analyzing their responses to queries from medical specialty associations.^13–15^ Other studies have tested LLMs’ performance in specific clinical tasks, often benchmarking their accuracy against that of human experts.^16–19^ Additionally, comparisons have been made between LLMs and traditional AI approaches,^20^ as well as search engines^21–23^ to assess their relative effectiveness. Despite these advances, there are still emerging opportunities and challenges in leveraging LLMs in healthcare.

Electronic health records (EHRs) have revolutionized healthcare by offering a comprehensive digital repository of a patient’s medical history, accessible to authorized providers across various healthcare settings. This seamless information sharing significantly enhances the quality, safety, and efficiency of patient care by integrating diverse data types, including medical history, diagnoses, medications, and test results. EHRs facilitate more accurate and timely decision-making, reduce the likelihood of medical errors, and contribute to improved patient outcomes. Additionally, they serve as an invaluable resource for healthcare research and quality improvement initiatives. However, the vast and complex datasets generated by EHRs present growing challenges for effective analysis and utilization.

While generative LLMs have been widely explored for healthcare data analysis, their application in real-world EHR data remains limited due to significant privacy concerns. For example, as of April 18, 2023, the use of ChatGPT with the widely used Medical Information Mart for Intensive Care (MIMIC) data has been explicitly prohibited,^24–26^ underscoring the need for Health Insurance Portability and Accountability Act (HIPAA)-compliant platforms to safely leverage LLMs on EHR data.^27^ Although several reviews have examined the broader use of generative LLMs in healthcare, there is a distinct lack of focused analyses on their application within EHR data for enhancing patient care in specific clinical tasks. To fill this gap, we conducted a systematic review that evaluates the effectiveness of various prompting and fine-tuning strategies in applying LLMs to specific clinical tasks. Additionally, we review the integration of multimodal data and its benefits, summarize the evaluation metrics used (e.g., confusion matrix, Likert scale) and evaluation purposes involved (e.g., correctness, completeness), and discuss future directions for the application of LLMs in clinical settings. This comprehensive analysis aims to provide critical insights and guide the advancement of patient care through these technologies.

## 2. Methods

### 2.1. Study Selection Process

**Figure 1.**
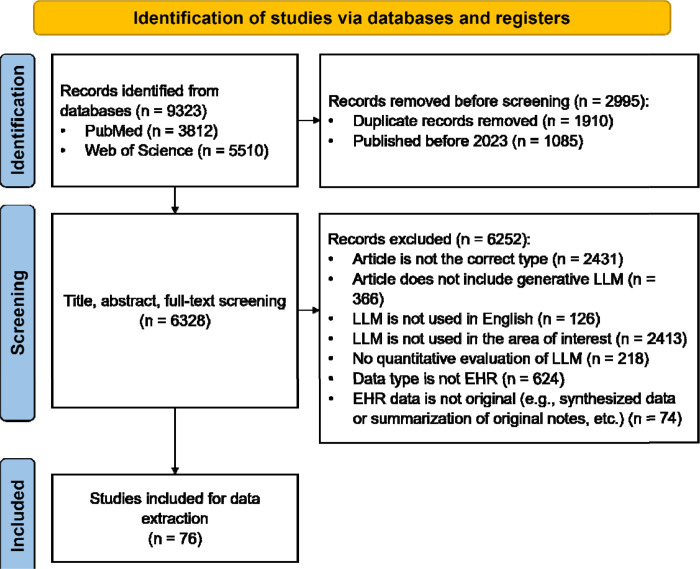
PRISMA Flow Chart for Eligibility Screening. We initially identified 9,323 studies from PubMed and Web of Science. After deduplication and excluding articles published before 2023, we included 6,328 studies for eligibility screening. Ultimately, 6,252 studies did not meet the inclusion criteria, leaving 76 studies for detailed analysis.

We adhered to the PRISMA guidelines for conducting our literature search (Figure 1).^28^ The process involved several key steps: a Boolean search, removal of duplicates, screening of studies, and data extraction. The Boolean search was conducted on May 19^th^, 2024, with search terms and restrictions determined through team discussions. Our search included LLM-related terms, such as “prompt engineering” and the names of various LLMs; the detailed query can be found in Supplementary Table S1. To focus on research articles presenting original data and quantitative results, we excluded certain article types, such as reviews. Given that the first release of ChatGPT was on November 30^th^, 2022, we included articles published from 2023 onward. Our search was conducted in PubMed and Web of Science, with only peer-reviewed articles included, while preprints were excluded.

### 2.2. Inclusion and Exclusion Criteria

Generative LLMs have been employed with various types of medical data, including but not limited to medical imaging, pharmaceutical data, public health data, genomics, biometric data, and EHR data. Our review specifically focuses on the application of LLMs to original EHR data, excluding studies using synthetic or summarized EHR data. For each included studies, we summarized the data size and data source.

The selection process began with the removal of duplicate articles, followed by a manual review of the deduplicated list. The exclusion criteria were as follows: (1) Articles that were not of the appropriate type (e.g., preprints, reviews, editorials, comments) were excluded. (2) Articles that did not involve generative LLMs were excluded; for example, those discussing chatbots that do not utilize LLMs were not considered. Our review specifically focuses on generative LLMs where prompt engineering can be applied. Although some encoder-based models like Longformer^3^, NYUTron^4^, GatorTron^5^ are powerful and widely used, studies involving only these encoder-based models were excluded. (3) Articles where the LLM was not used for English-language communication were not included. (4) Articles where the LLM application was unrelated to patient care (e.g., LLMs used for passing exams or conducting research) were excluded. (5) Articles that lacked quantitative evaluation (e.g., those that only presented communication records with ChatGPT) were excluded. (6) Articles that did not involve EHR data were excluded. (7) Articles where the EHR data used was not original (e.g., synthetic or summarized data) were excluded.

During the eligibility screening process, two reviewers initially screened a set of 50 identical articles. If the agreement rate was above 90%, the reviewers proceeded to independently screen the remaining articles. If not, they discussed and screened an additional 50 articles until the agreement reached 90%.

### 2.3. Data Extraction and Statistical Analysis

For the included studies, we extracted various categories of information, as detailed in Table 1. This includes data-related information, clinical information, LLM-specific details, evaluation metrics, and identified challenges. The extracted data encompasses key aspects such as the nature and source of the data, the clinical context in which the LLM was applied, the specific LLM models and techniques used, the methods of evaluation employed, and the current challenges faced in these applications. Additionally, we provided detailed explanations of existing techniques for prompt engineering, generative LLM fine-tuning, and multimodal data integration in the Supplementary Material.

#### 2.3.1. Overview of Included Studies

We extracted details on data size and data source from the included studies. Data size refers to the number of samples used in each study, while data source indicates the origin of the data, which could be from a specific hospital or a publicly available EHR dataset, such as MIMIC.^25,26^ We extracted information on clinical specialties from each included study, and the distribution of clinical specialties was summarized using a pie chart. Specialties represented in less than 5% of the studies were consolidated into a single category. Additionally, we used bar charts to represent the frequency of used prompting strategies, fine-tuning approaches, studied LLMs, and evaluation purposes.

#### 2.3.2. Prompt Engineering

Regarding prompting methods, we documented the specific prompting strategy used, the clinical tasks they were applied to, the performance and the quantitative impact of each prompting approach on the performance of these tasks.

#### 2.3.3. Fine-Tuning

In terms of fine-tuning, we extracted information on the base models that were fine-tuned, the specific fine-tuning methods employed, the hardware used for the fine-tuning process, the performance and the quantitative effects on clinical task performance.

#### 2.3.4. Multimodal Data Integration

For studies involving the application of LLMs to multimodal EHR data, we summarized the data modalities involved, the methods used for data integration, and the quantitative impact of multimodal integration on performance.

#### 2.3.5. Evaluation Matrices and Purposes

Researchers employ various evaluation purposes depending on the specific clinical task when assessing LLM performance. For example, in clinical decision-making, the emphasis may be on the accuracy and completeness of the LLM’s output, whereas for clinical note summarization or simplification, readability and conciseness are primary evaluation criteria. Given that LLM responses may be used in clinical settings (e.g., providing clinical advice to patients), additional factors such as the potential harmfulness of the output and the level of empathy conveyed are also critical aspects of performance evaluation.

We summarized the terms from the included studies to represent evaluation purposes, consolidating terms with the same meaning (e.g., reliability and stability) into a single category. A bar chart was used to illustrate the frequency of each evaluation purpose. For each evaluation metric, we summarized its purpose and the best reported value in relation to the corresponding clinical tasks. Additionally, for NLP metrics that assess the similarity between the LLM’s output and the ground truth, we identified correlated metrics that require human judgment for validation.

#### 2.3.6. Generative LLM Challenges

An introduction to the existing challenges is provided in the Supplementary Material. This review summarizes the current challenges of applying generative LLMs to EHR data, including bias, common errors, hallucinations, and other issues identified in the included studies.

## 3. Results

### 3.1. Study Selection Results

As illustrated in Figure 1, our Boolean search initially yielded 9,323 articles. After removing 1,910 duplicates and excluding 1,085 articles published before 2023, we had 6,328 articles remaining for screening. Following a thorough screening of titles, abstracts, and full articles, we ultimately included 76 eligible studies for further analysis.

### 3.2. Analysis Result

#### 3.2.1. Overview of Included Studies

**Figure 2.**
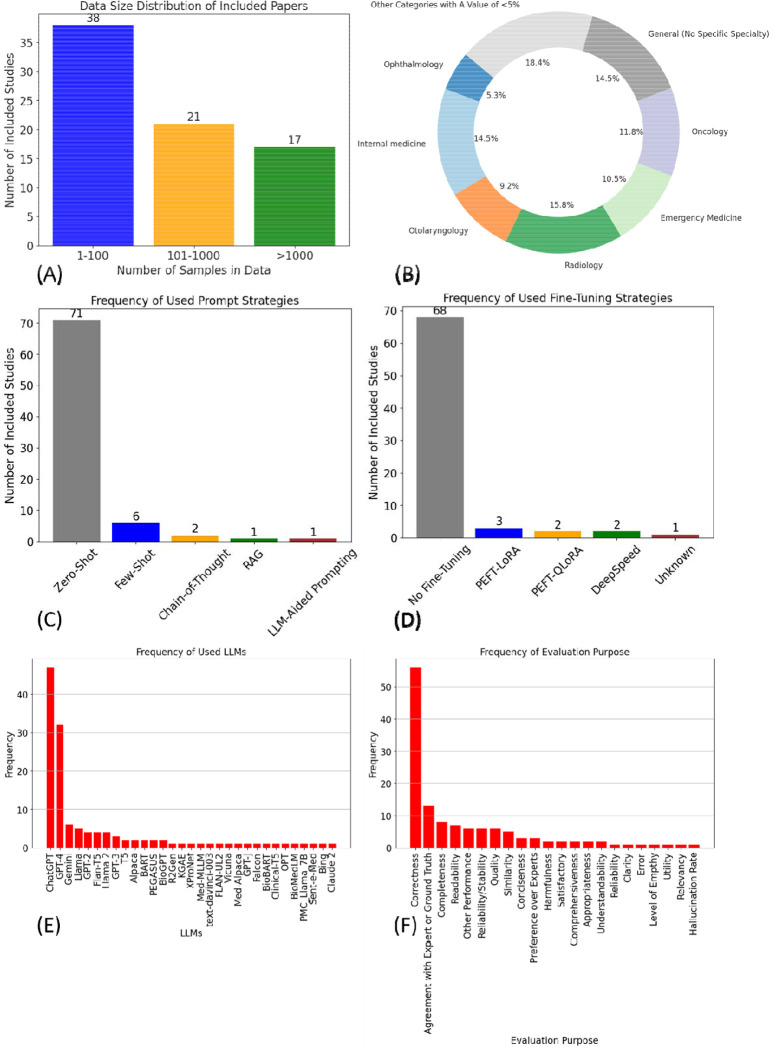
Result Summarization. (A) illustrates the data size distribution of the included studies, with the majority (38 out of 76, 50%) comprising less than 100 samples. (B) depicts the distribution of clinical specialties, where radiology emerges as the most frequently studied specialty, representing 15.8% of the studies. (C) shows the frequency of prompting strategies used, with few-shot prompting (N=6) being the most popular among the advanced strategies. (D) presents the frequency of fine-tuning approaches, with PEFT-LoRA (N=3) identified as the most commonly employed fine-tuning method. (E) is a bar plot displaying the frequency of different LLMs used in the studies, with ChatGPT leading as the most frequently utilized model, appearing in 48 studies. (F) highlights the frequency of evaluation purposes across the studies, with correctness being the most commonly assessed factor, evaluated in 57 studies.

The distribution of data sizes is shown in Figure 2 (A). Detailed information on data size and data source is provided in Supplementary Table S2. We found that 38 studies (50.0%) had a data size of less than 100, 21 studies (27.8%) had a data size between 100 and 1,000, and 17 studies (21.5%) had a data size greater than 1,000. The distribution of clinical specialties is shown in

Figure 2 (B). The top three clinical tasks identified are radiology (15.8%), general—no specific specialty (14.5%), and internal medicine (14.5%). Detailed information on the clinical tasks of each included study is provided in Supplementary Table S2.

Figure 2 (C) shows the distribution of prompting strategies used across the included studies. Zero-shot prompting was by far the most commonly employed strategy, with 71 out of 76 studies utilizing this approach. Few-shot prompting was used in six studies, while chain-of-thought was applied in two. Other strategies, such as Retrieval-Augmented Generation (RAG) and LLM-Aided Prompting, were also studied but to a lesser extent. Additionally, four studies combined multiple prompting strategies in their approach.

Figure 2 (D) presents the frequency of different fine-tuning methods used. A significant majority, comprising 68 studies, did not involve any fine-tuning. Of the remaining studies, three employed Parameter-Efficient Tuning (PEFT) using Low Rank Adaptation (LoRA), two used a combination of PEFT and Quantization-LoRA (QLoRA), two implemented DeepSpeed, and one study did not disclose the specific fine-tuning technique used.

Figure 2 (E) provides an overview of the frequency with which different generative Large Language Models (LLMs) were used in the studies. ChatGPT was the most frequently utilized model, appearing in 48 studies (63.2%). This was followed by GPT-4, which was used in 32 studies (42.1%). Google Gemini was the next most common LLM, appearing in six studies (7.9%).

Figure 2 (F) details the frequency of evaluation purposes across the studies. The most frequent evaluation purpose was correctness, with 57 instances. This was followed by agreement with expert opinion or ground truth (12 instances) and completeness (8 instances). Other evaluation purposes, such as reliability, comprehensiveness, and hallucination rate, were less frequently examined.

#### 3.2.2. Prompting Methods

Table 1 summarizes the findings on prompting methods used in the included studies. Nine studies employed advanced prompting techniques, while the remaining 67 studies used zero-shot prompting only. Four studies specifically discussed strategies for crafting zero-shot prompts to enhance LLM performance on specific clinical tasks. Among the advanced prompting techniques, three studies used few-shot prompting, two studies employed chain-of-thought prompting, two studies utilized soft prompting, one study involved RAG, and one study used another LLM to assist with prompt generation.

All studies that used advanced prompting techniques reported improvements in LLM performance due to prompt engineering, though the significance of these improvements varied depending on the clinical task. For instance, one study found that a combination of few-shot prompting, chain-of-thought, and RAG increased the LLM’s F1 score by 5% to 15% on a subset of 100 reports when detecting speech recognition errors in radiology reports^29^ Another study combined soft prompting with LLM-aided prompting (using an LLM to help generate prompts) for clinical note summarization and found that LLM-aided prompting improved ROUGE-1 by 1% to 3%, ROUGE-2 by 2% to 4%, and ROUGE-L by 1% to 2%, while soft prompting reduced response variability by up to 43%.^30^

#### 3.2.3. Fine-Tuning Methods

Table 2 summarizes the studies that fine-tuned LLMs for specific clinical domains or tasks. Of the 76 included studies, only 8 (10.5%) involved LLM fine-tuning. Regarding the fine-tuning methods, three studies used parameter-efficient fine-tuning (PEFT) with Low-Rank Adaptation (LoRA)^31^, two used PEFT-Quantized LoRA (QLoRA),^32^ two utilized DeepSpeed^33^ for full parameter tuning, and one study did not specify the fine-tuning technique.

While six of the eight studies reported that fine-tuning improved performance on clinical tasks, three studies noted potential drawbacks of fine-tuning, such as 1) catastrophic forgetting^34^ and 2) low relevance between the fine-tuning data and the LLM’s application domain or task.^35,36^ Additionally, it was observed that a smaller fine-tuned model can sometimes outperform a larger base model in specific domains and tasks. For example, in differential diagnosis for PICU patients, the fine-tuned Llama-7B achieved an average quality score of 2.88, while the Llama-65B without fine-tuning achieved an average quality score of 2.65 out of 5.00^37^

#### 3.2.4. Multimodal Data Fusion for LLM

Two of the included studies utilized multimodal data. In one study, different types of data were encoded and fused within the AI model itself after encoding each input data modality.^38^ The other study converted various data modalities into text format before feeding the text into the model.^39^ Integrating multimodal data was shown to enhance overall performance. For instance, a Llama model trained on multimodal data achieved a higher macro F1 score (22.3%) compared to a Llama model trained solely on medical notes (macro F1 = 21.8%) for disease diagnosis.^38^ Similarly, using multimodal data for pre-training and fine-tuning LLMs led to better performance in diagnosing COVID-19 (accuracy = 90.3% vs. 84.1%) and prognosticating COVID-19 (accuracy = 92.8% vs. 94.9%) when compared to using text-only data.^39^ Notably, the study pre-trained the LLM on Delta COVID-19 data, fine-tuned it on 1% of Omicron data, and then evaluated it on the remaining 99% of Omicron data. This also suggests that multimodal LLMs can effectively handle scenarios where training data is scarce, such as in diagnosing or prognosticating rare diseases.

#### 3.2.5. Evaluation Matrices and Purposes

Figure 1(D) presents the statistics on the evaluation methods used in the included studies. A total of 22 evaluation purposes were identified. The three most frequently used evaluation purposes were correctness (employed in 56 studies), agreement with experts or ground truth (used in 12 studies), and completeness, reliability/stability, and readability (each used in 7 studies). For assessing accuracy, confusion matrix-based metrics were the most employed.

Table 3 provides a summary of all evaluation metrics used in the included studies. A total of 55 different evaluation metrics were identified, with 35 of them being NLP metrics that measure the similarity between the generative LLM’s response and the gold standard response. Four studies used Spearman’s correlation to examine the relationships between evaluation metrics.^35,36,40^ The findings were as follows: 1) The Artificial Intelligence Performance Instrument (AIPI) correlated with the Ottawa Clinic Assessment Tool (OCAT) when managing cases in otolaryngology–head and neck surgery (ρ = 0.495); 2) BERTScore correlated with the quality score derived from a Likert scale when generating impressions for whole-body PET reports (ρ = 0.474); 3) BERTScore correlated with conciseness when summarizing patient questions and progress notes; and 4) when generating concise and accurate layperson summaries of musculoskeletal radiology reports, BERTScore and MEDCON Score correlated with correctness (ρ = 0.17), and BLEU correlated with completeness (ρ = 0.225).

#### 3.2.6. Challenges for Applying in Real Clinical Settings

##### 3.2.6.1. Bias

Among the included studies, only two specifically examined the bias of LLMs. One study reported that ChatGPT did not exhibit biases related to demographic factors such as age and gender when making imaging referrals.^41^ However, the other study found that male patients received more appropriate responses than female patients, indicating a potential gender bias in how ChatGPT processes information.^42^

##### 3.2.6.2. Common Errors

Several studies highlighted common errors made by LLMs. For instance, multiple studies pointed out that the LLM made more errors when diagnosing uncommon cases.^43^ GPT-4 was found to sometimes miss important details when converting radiological reports into a structured format.^44^ Additionally, multiple studies indicated that LLMs were not proficient in recommending appropriate treatments or examinations^30,45^. One study showed that ChatGPT often provided unnecessary treatments for 55% of patients with head and neck cases^46^, and for 67%–90% of such patients in other instances.^47^ Another study reported that unnecessary treatments were recommended by ChatGPT for 55% of patients with positive blood cultures,^48^ and ChatGPT was more likely to suggest additional treatments compared to physicians (94.3% vs. 73.5%, p<0.001).^49^ For rhinologic cases, the accuracy of GPT-4 in suggesting treatment strategies was only 16.7%^50^

Several studies also found that LLMs performed poorly when triaging patients. For example, when providing triage for maxillofacial trauma cases, Gemini inadequately proposed intermaxillary fixation and missed the necessity of teeth splinting in another case.^51^ In the emergency department, ChatGPT provided unsafe triage in 41% of cases.^52^ Furthermore, LLMs may omit critical information in patient history. When tasked with improving the readability of clinical notes, LLMs were found to omit the history of present illness and procedures in 52.1% of cases^53^ ChatGPT, relying on static data, lacks the ability to assess individual patient history when diagnosing conditions like bacterial tonsillitis.^54^ Additionally, studies found that patients had difficulty understanding ChatGPT’s responses, and the readability of ChatGPT-generated responses to patient-submitted questions was not as good as those produced by dermatology physicians.^55^ ChatGPT also struggles with diagnosing complex diseases due to ambiguous symptoms.^54,56^ Two studies noted that ChatGPT might overlook compositional information and adjacent relationships of nodules when diagnosing tumor-related diseases^57,58^

##### 3.2.6.3. Hallucinations

LLMs can sometimes generate hallucinations, producing content that is inaccurate or fabricated. When identifying clinical phenotypes within the complex notes of rare genetic disease patients, GPT-J may invent Human Phenotype Ontology (HPO) IDs, even after fine-tuning and using few-shot prompting.^59^ In another instance, when identifying confidential content in clinical notes, 87% of the 306 excerpts proposed by ChatGPT from a note containing confidential information included hallucinations.^60^ Additionally, when extracting the clinical factor of neoadjuvant chemotherapy status in breast cancer patients, ChatGPT provided a yes or no answer despite the pathology report lacking any relevant information.^61^ While summarizing clinical letters, ChatGPT occasionally inserted sentences that were not present in the original letter, such as “please know that we are here to support you every step of the way” and “your expertise and insights are invaluable”.^62^ ChatGPT has also been known to fabricate patient names when generating structured thyroid ultrasound reports from unstructured ultrasound reports.^58^ Moreover, when improving the readability of radiology reports, ChatGPT incorrectly stated that a patient had a lateral ligament complex tear when the lateral ligament complex was intact or claimed there was no fracture of the lateral malleolus when a fracture was indeed present.^63^

##### 3.2.6.4. Other Challenges

Three included studies noted that patients felt uncomfortable with ChatGPT’s impersonal tone during consultations, and they often found it difficult to understand ChatGPT’s responses.^55,62,64^

## 4. Discussion

Recent publications on generative LLMs in healthcare underscore their evolving role and the wide range of potential applications. Numerous reviews have been published to summarize the field’s development, with a general consensus that LLMs hold significant promise in clinical settings, assisting physicians in tasks such as answering patient questions and improving the readability of medical documents. However, challenges remain in applying LLMs in clinical environments. Omiye et al. reviewed LLM applications in medicine and identified major challenges, including bias, data privacy concerns, and the unpredictability of outputs.^9^ Clusmann et al. emphasized that hallucinations are a significant obstacle,^10^, while Acharya et al. attempted to address this issue by fine-tuning LLMs, only to find that this process led to the loss of previously acquired knowledge.^34^ Additionally, Wornow highlighted the lack of benchmarks and standardized evaluation techniques necessary to ensure LLM reliability in real clinical settings.^11^ Unlike existing reviews, our study extends previous work by summarizing the techniques, challenges, and opportunities for applying LLMs to real EHR data to improve patient care—an area where corresponding studies remain rare due to privacy concerns.

Our review found that out of the 76 included studies, 67 relied on zero-shot prompting. Among the studies that employed a specific prompting strategy, only four evaluated its effectiveness, and all four reported that using prompting strategies improved performance. For instance, one study noted that soft prompting reduced the variability of LLM outputs when summarizing clinical notes.^30^ However, recent research has suggested that prompting strategies, such as few-shot prompting, do not always lead to performance improvements.^27,65^ This may be due to the fact that prompting strategies can increase the length of a prompt, and a longer prompt might negatively impact the LLM’s performance.^66^ Furthermore, the use of prompting strategies is often limited by the maximum length constraints of an LLM. Therefore, further testing of prompting strategies in specific clinical tasks and specialties is necessary to validate their effectiveness in real clinical settings.

Unlike prompting strategies, fine-tuning an LLM enables it to fully leverage all labeled training data without concerns about maximum length limits. However, fine-tuning proprietary LLMs (e.g., ChatGPT and GPT-4) is often restricted, and fine-tuning open-source LLMs requires expensive hardware. Fortunately, one included study demonstrated that fine-tuning a smaller language model can outperform an unfine-tuned large model.^37^ Techniques like LoRA and QLoRA allow researchers to fine-tune LLMs with more affordable hardware,^31,32^ and the DeepSpeed algorithm can accelerate the fine-tuning process.^33^ It’s important to note, however, that fine-tuning may not enhance performance if the fine-tuning dataset lacks sufficient text relevant to the specific domain and task.^35^ For instance, if the goal is to optimize LLM performance in analyzing PET reports, it would be more effective to fine-tune the model using a large corpus of PET reports rather than a mix of different clinical notes. Therefore, in clinical settings, we recommend fine-tuning a smaller, open-source language model with a domain- and task-specific corpus to achieve better results in specific domains and tasks.

Incorporating multimodal clinical data enhances the performance of clinical decision support systems and enables LLM-based support for rare diseases.^39^ Notably, several studies mentioned that LLMs struggle with handling rare diseases, likely due to the limited information about rare conditions in the training data. We also observed that only two of the included studies utilized multimodal data, indicating a need for more research in the future focused on leveraging LLMs and multimodal EHR data to address challenges in rare disease diagnosis and management.

Our review indicates a pressing need for standardized evaluation metrics and solutions to reduce the labor-intensive nature of human evaluation. We found that different studies often use varying metrics to achieve the same evaluation goals, highlighting the necessity of establishing standardized metrics for each evaluation purpose to benchmark performance consistently. Although expert evaluation is considered the gold standard, it is impractical for physicians to thoroughly review all LLM outputs for performance evaluation.^35^ As data sizes increase, manual review becomes increasingly labor-intensive, costly, and time-consuming. This challenge may also explain why 50% of the included studies used a small data size of less than 100 samples. Fortunately, some studies have identified correlations between automated similarity metrics and human subjective evaluation metrics. For instance, BLEU scores showed a Spearman’s correlation coefficient of 0.225 with physicians’ preferences for completeness when summarizing clinical texts.^36^ Therefore, developing standardized objective metrics for each evaluation purpose is crucial for ensuring fair and effective evaluations. Additionally, further investigation is needed to explore how automated evaluation metrics can replace human subjective evaluation, particularly when dealing with large datasets.

Overall, while ChatGPT and similar LLMs present innovative potential in medical diagnostics and patient interaction, significant challenges and biases persist. Although only a limited number of studies have examined biases in large language models, there is evidence of gender-related bias in ChatGPT’s responses. For instance, one study found no bias in imaging referrals related to age or gender,^60^ while another highlighted a gender bias, with male patients receiving more appropriate responses than female patients.^61^ This finding underscores the need for ongoing evaluation and mitigation of biases in LLMs to ensure equitable and unbiased healthcare information for all users. Additionally, these models often struggle with diagnosing uncommon cases,^62^ accurately converting radiological reports,^63^ and recommending appropriate treatments.^51,64–48^ The tendency to suggest unnecessary treatments and the high rate of unsafe triage decisions^69, 70^ further highlight the risks associated with relying on LLMs in clinical settings. LLMs may also omit critical patient history details^71, 72^ and provide responses that are difficult for patients to understand.^73^ Their inadequacies in handling complex diseases and ambiguous symptoms,^72, 74^ as well as the potential for overlooking essential information,^75, 76^ suggest that LLMs currently lack the reliability needed for high-stakes medical decision-making. These findings emphasize the need for continuous improvement and careful integration of LLMs into healthcare to mitigate risks and enhance patient safety.

The findings regarding hallucinations in generative LLMs like GPT-J and ChatGPT highlight a critical issue that limits the reliability and safety of these models in clinical settings. Hallucinations, which involve the generation of fabricated or incorrect information, are particularly concerning when LLMs are used for tasks requiring high accuracy and trust, such as in healthcare. For example, GPT-J’s tendency to create fictitious Human Phenotype Ontology (HPO) IDs when addressing rare genetic diseases suggests that even advanced fine-tuning and prompting techniques may not fully eliminate the risk of hallucinations.^77^ This issue not only compromises the accuracy of diagnoses but also risks misleading healthcare providers who might rely on these outputs in decision-making processes.

Moreover, ChatGPT has exhibited similar issues across various medical applications. The model has been shown to insert non-existent information into clinical notes and summaries, fabricating phrases intended to convey support or even creating fictitious patient names when generating structured reports.^78–80^ These errors are far from benign; they have the potential to cause real harm, especially if clinicians act on incorrect information. The implications of these hallucinations are significant. For instance, misstating the condition of the lateral ligament complex or incorrectly identifying the presence of fractures can lead to inappropriate treatment plans and delayed care.^81^ Such inconsistencies and inaccuracies call into question the reliability of LLMs in clinical environments, emphasizing the need for their cautious use, particularly in high-stakes situations.

Beyond technical inaccuracies, the impersonal tone of ChatGPT’s responses and the challenges patients face in understanding these responses further diminish the effectiveness of LLMs in patient interaction.^73, 80, 82^ The lack of empathy and clarity in communication can erode patient trust and satisfaction, both of which are critical components of effective healthcare delivery. While LLMs hold significant promise for enhancing healthcare through automation and data processing, the risks posed by hallucinations and communication challenges must be addressed. Until these issues are resolved, the integration of LLMs into healthcare should proceed with caution, ensuring that human oversight remains central to patient care.

Our review has several strengths and weaknesses. Given the rapid development in the field, the volume of articles on LLMs in healthcare is substantial. We identified studies published since 2023 from two databases (PubMed and Web of Science) and thoroughly screened each article based on our eligibility criteria. Every included study was analyzed in depth, and we provided detailed summaries. However, a limitation of our review is that our Boolean search was conducted in May 2024, so studies published online after this date were not included.

## 5. Conclusion

We conducted a systematic literature review to summarize articles that use LLMs to analyze real EHR data for improving patient care. We found that the application of prompt engineering and fine-tuning techniques is still relatively rare. Additionally, only two studies utilized LLMs with multimodal EHR data, and they demonstrated that incorporating multimodal data can enhance decision-making performance and enable more accurate diagnoses of rare diseases. Several limitations of LLMs were identified, making them currently unsuitable for widespread use in clinical practice. These limitations include the lack of standardized evaluation methods, impersonal tone and low readability in responses to patient questions, and the presence of biases and hallucinations in generated responses.

Future research should focus on exploring more prompt engineering and fine-tuning approaches tailored to specific clinical domains and tasks to optimize their use. Additionally, important future directions include standardizing evaluation metrics, mitigating bias and hallucinations, and applying LLMs to multimodal data to further improve their performance.

## Supplementary Material

Supplement 1

Supplement 2

## Figures and Tables

**Table:** 

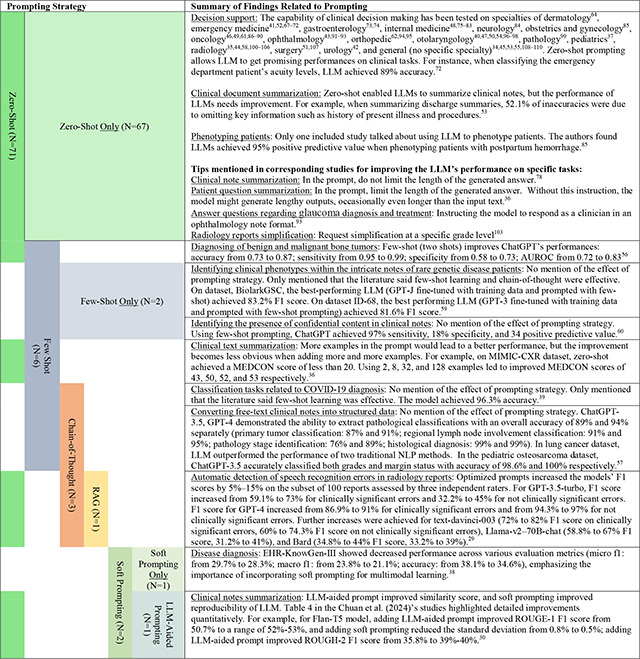

**Table 2.** Eight Included Studies with LLM Fine-Tuning.

LLMs That Was Fine-Tuned	Fine-Tuning Algorithm	Fine-Tuning Hardware	Summary of Findings Related to Fine-Tuning
Llama	Full Parameter - DeepSpeed	2*NVIDIA A100 GPUs	Disease diagnosis: No mention regarding the comparison of fine-tuned model and the original model. Fine-tuned Llama achieved 34.9% accuracy, 22.3% macro f1 score, and 28.7% micro f1 score.^38^
Llama	PEFT-LoRA	1*Nvidia RTX A6000 GPU	Predicting diagnosis-related group for hospitalized patients: No mention regarding the comparison of fine-tuned model and the original model. DRG-LLaMA -7B model exhibited a noteworthy macro-averaged F1 score of 0.327, a top-1 prediction accuracy of 52.0%, and a macro-averaged Area Under the Curve (AUC) of 0.986.^109^ 1) A larger base model led to better fine-tuned performance. The best diagnosis accuracy of the fine-tuned Llama-13B achieved 54.6%, while that of the fine-tuned 7B model achieved 53.9%. 2) Longer input context from the fine-tuning data led to better performance. For fine-tuned Llama-13B, when the max input token size was 340, the best diagnosis accuracy was 49.9%, but the accuracy was increased to 54.6% when the max token size was 1024.
Llama 2; FLAN-T5; FLAN-UL2; Vicuna Alpaca	PEFT-QLoRA	1*NVIDIA Quadro RTX 8000	Clinical text summarization: Fine-tuned FLAN-T5 improved MEDCON score from 5 to a range of 26–69 on four datasets.^36^ 1) QLoRA FLAN-T5 was the best-performing fine-tuned open-source model. It achieved a MEDCON score of 59 on Open-i data, 38 on MIMIC-CXR data, 26 on MIMIC-III data, and 46 on patient questions data. 2) QLoRA typically outperformed ICL with the better models (FLAN-T5 and Llama-2); given a sufficient number of in-context examples (from 1 to 64), however, all models surpassed even the best QLoRA fine-tuned model, FLAN-T5, in at least one dataset. 3) An LLM fine-tuned with domain-specific data performed worse than the original model. For example, when Alpaca achieved a BLEU value of 30, Med-Alpaca only reached 20. This highlights a distinction between domain adaptation and task adaptation.
GPT-3; GPT-J; Falcon; Llama	PEFT-QLoRA	Open AI’s Cloud Resources	Phenotype recognition in clinical notes: No quantitative comparison between models before and after fine-tuning. Fine-tuned GPT-3 achieved the best performance of 81.6% F1 score on one dataset, and fine-tuned GPT-J performed the best on the other dataset (83.2% F1 score) ^59^
BART; PEGASUS; T5; FLAN-T5; BioBART; Clinical-T5; GPT2; OPT; Llama; Alpaca	PEFT-LoRA for Llama and Alpaca; full parameter tuning for other models.	At least two NVDIA A100 GPUs	Generating personalized impressions for whole-body PET reports: Biomedical domain pretrained LLMs did not outperform their base models. Specifically, the domain-specific fine-tuned BART model reduced the accuracy from 75.3% to 73.9%. This could be attributed to two reasons. First, our large training set diminished the benefits of medical-domain adaptation. Second, the corpora, such as MIMIC-III and PubMed, likely had limited PET-related content, making pretraining less effective for our task.^35^
Llama 2	No mention	No mention	Predicting opioid use disorder (OUD), substance use disorder (SUD), and Diabetes: Fine-tuned Llama 2 achieved 92%, 93%, 74%, and 88% AUROC on four datasets for predicting SUD. Fine-tuned Llama 2 achieved 95%, 72%, 73%, and 98% AUROC on four datasets for predicting OUD. Fine-tuned Llama 2 achieved 88%, 76%, 64%, and 94% AUROC on four datasets for predicting diabetes.^34^ 1) An experiment of changing instructions suggests that fine-tuning on our datasets might have induced catastrophic forgetting particularly when dealing with a large volume of data. 2) Fine-tuned Llama 2 outperformed Llama 2 without fine-tuning on diabetes prediction (AUROC increased from 50% to 88%).
Llama 2–7B; BioGPT-Large	Full Parameter - DeepSpeed	4*A40 Nvidia GPUs	Differential Diagnoses in PICU Patients: Fine-tuned model outperformed original model, but a smaller LM fine-tuned using domain-specific notes outperformed much larger models trained on general-domain data.^64^ Specifically: 1) Fine-tuned Llama-7B achieved an average quality score of 2.88, while Llama-65B without fine-tuning achieved an average quality score of 2.65. 2) Fine-tuned BioGPT-Large had an average score of 2.78, while BioGPT-Large without fine-tuning had a mean score of 2.02
BART; GPT; MedLM	PEFT-LoRA	No mention	Early detection of gout flares based on nurses’ chief complaint notes in the emergency department: No comparison between models before and after fine-tuning. Fine-tuned BART model (BioBART) performed the best, which achieved 0.73 and 0.67 F1 score on datasets GOUT-CC-2019-CORPUS and GROUT-CC-2020-CORPUS.^71^

**Table 3.** Summary of Evaluation Matrices.

General Matrices						
Evaluation Matrices	Evaluation Purpose	Best Reported Performance		Clinical Task		Clinical Specialty
Confusion Matrices-Based Scores	Correctness	100%		Diagnosing glaucoma based on specific clinical case descriptions^43^		Ophthalmology
				Generating radiology reports from concise imaging findings^100^		Radiology
				Accelerating review of historic echocardiogram reports^77^		Internal Medicine
				Interpret symptoms and management of common cardiac conditions^79^		Internal Medicine
				The diagnosis management of bacterial tonsillitis^54^		Otolaryngology
				Classifying margin status for lung cancer^57^		Oncology
Average Word Count Reduction Percentage + Recall	Balance Between Conciseness and Completeness	Average Word Count Reduction Percentage=47% when Recall=90%		Summarizing radiology reports into structured format^44^		Radiology
Self-Designed Human Evaluation (e.g., Likert-Scale)	Correctness	89.6%		Generating concise and accurate layperson summaries of musculoskeletal radiology reports^101^		Radiology
	Completeness	94.1%		Generating concise and accurate layperson summaries of musculoskeletal radiology reports^101^		Radiology
	Conciseness	12%		Summarizing patient questions and progress notes^36^		No specific specialty
	Harmfulness*	2%		Proposing a comprehensive management plan (suspected/confirmed diagnosis, workup, antibiotic therapy, source control, follow-up) for patients with positive blood cultures^48^		Internal Medicine
	Readability	80%		Generating radiology reports from concise imaging findings^100^		Radiology
	Quality	89%		Impression generation for whole-body PET reports^35^		Radiology
	Appropriateness	58.5%		Diagnosing and suggest examinations/treatments for urology patients (subgroups that had the best performance: non-oncology)^42^		Urology
	Satisfactory	80%		Proposing a comprehensive management plan (suspected/confirmed diagnosis, workup, antibiotic therapy, source control, follow-up) for patients with positive blood cultures^48^		Internal Medicine
	Reliability/Stability	70%		Predicting treatments for patients with aortic stenosis^83^		Internal Medicine
	Preference over Human	81%		Summarizing clinical text^36^		No specific Specialty
	Level of Empathy	61.4%		Generating high-quality responses to patient-submitted questions in the patient portal^64^		Dermatology
	Hallucination Rate*	4%		Improving the readability of foot and ankle orthopedic radiology reports^106^		Radiology
	Utility	81.6%		Impression generation for whole-body PET reports^35^		Radiology
	Relevancy	40%		Simplifying radiological MRI findings of the knee joint^105^		Radiology
Artificial Intelligence Performance Instrument (AIPI)	Other Performance	15.1/20.0		Managing cases in otolaryngology–head and neck surgery^40^		Otolaryngology
QAMAI Tool	Other Performance	18.4/30		Providing Triage for Maxillofacial Trauma Cases		Surgery
Ottawa Clinic Assessment Tool	Other Performance	3.88/5.00		Recommending differential diagnosis for laryngology and head and neck (Recommending differential diagnosis) cases^47^		Otolaryngology
DISCERN	Quality	15/35		Diagnosing and suggest examinations/treatments for urology patients (subgroups that had the best performance: oncology, emergency, and male)^42^		Urology
Root Mean Square Error	Error	2.96		Measuring the angle of correction for high tibial osteotomy^95^		Orthopedic
Flesch Reading Ease	Readability	72.7%		Improving the readability of foot and ankle orthopedic radiology reports^63^		Radiology
Flesch-Kincaid Reading Grade Level*	Readability	6.2		Summarizing discharge summary^53^		No specific specialty
Average of Gunning Fog, Flesch–Kincaid Grade Level, Automated Readability, Coleman–Liau*	Readability	7.5		Summarizing X-Ray report^103^		Radiology
Patient Education Materials Assessment Tool	Understandability	81%		Summarizing discharge summary^53^		No specific specialty
Cohen’s Kappa	Reliability/Stability	1.0		Head and neck oncological board decisions: deciding on neoadjuvant chemotherapy and chemoradiotherapy treatment		Oncology
	Agreement with Expert or Ground Truth	0.727		Predicting the dichotomized modified Rankin Scale (mRS) score at 3 months post-thrombectomy^84^		Neurology
Cronbach’s α	Agreement with Expert or Ground Truth	0.754		Managing otolaryngology cases^96^		Otolaryngology
Mann-Whitney U test	Agreement with Expert or Ground Truth	0.770		Providing number of additional examinations when managing otolaryngological cases^40^		Otolaryngology
Spearman’s Coefficient	Reliability/Stability	0.999		Considering the patient’s symptoms and physical findings reported by practitioners when managing otolaryngology cases^96^		Otolaryngology
Percentage of Getting the Same Response to Identical Queries	Reliability/Stability	100%		Predicting hemoglobinopathies from a patient’s laboratory results of CBC and ferritin values^82^		Internal Medicine
Agreement Percentage	Agreement with Expert or Ground Truth	80%		Determining disease severity for acute ulcerative colitis presentations in the setting of an emergency department^75^		Gastroenterology
Global Quality Scale	Quality	4.2		Analyzing retinal detachment cases and suggesting the best possible surgical planning^92^		Ophthalmology
Fleiss Kappa	Reliability/Stability	0.786		Colonoscopy recommendations for colorectal cancer rescreening and surveillance^74^		Gastroenterology
Similarity Measurements for Generative NLP Models						
Evaluation Matrices	Correlated Evaluation (If Reported) Measured by Spearman’s Coefficient			Performance		
	Reported Coefficient	Evaluation and Task	Clinical Specialty	Best Reported Value	Task	Clinical Specialty
BLEU	0.412	Quality score of Impression generation for whole-body PET reports^35^	Radiology	24.7	Impression generation for whole-body PET reports^35^	Radiology
	0.225	Completeness of clinical text summarization^36^	No Specific Specialty			
	0.125	Correctness of clinical text summarization^36^				
	0.075	Conciseness of clinical text summarization^36^				
BLEU-2				74.5	Generating a comprehensive and coherent medical report of a given medical image from COVID-19 data^39^	Internal Medicine
BLEU-3				67.8		
BLEU-4				63.2		
ROUGE-1	0.402	Quality score of Impression generation for whole-body PET reports^35^	Radiology	57.29	Clinical notes summarization^30^	No Specific Specialty
ROUGE-2	0.379	Quality score of Impression generation for whole-body PET reports^35^	Radiology	44.32	Clinical notes summarization^30^	No Specific Specialty
ROUGE-L	0.22	Completeness of clinical text summarization^36^	No Specific Specialty	68.5	Generating a comprehensive and coherent medical report of a given medical image from COVID-19 data^39^	Internal Medicine
	0.16	Correctness of clinical text summarization^36^				
	0.19	Conciseness of clinical text summarization^36^				
	0.398	Quality score of Impression generation for whole-body PET reports^35^	Radiology			
BERTScore-Precision				86.57	Clinical notes summarization^30^	No Specific Specialty
BERTScore-Recall				87.14		
BERTScore-F1	0.18	Completeness of clinical text summarization^36^	No Specific Specialty	89.4	Summarizing longitudinal aneurysm reports^102^	Radiology
	0.18	Correctness of clinical text summarization^36^				
	0.24	Conciseness of clinical text summarization^36^				
	0.407	Quality score of Impression generation for whole-body PET reports^35^	Radiology			
MEDCON	0.125	Completeness of clinical text summarization^36^		64.9	Clinical text summarization^36^	No Specific Specialty
	0.175	Correctness of clinical text summarization^36^				
	0.15	Conciseness of clinical text summarization^36^				
CIDEr	0.194	Quality score of Impression generation for whole-body PET reports^35^	Radiology	97.5	Generating a comprehensive and coherent medical report of a given medical image from COVID-19 data^39^	Internal Medicine
BARTScore+PET	0.568			−1.46	Impression generation for whole-body PET reports^35^	Radiology
PEGASUSScore+PET	0.563			−1.44		
T5Score+PET	0.542			−1.41		
UniEval	0.501			0.78		
BARTScore	0.474			−3.05		
CHRF	0.433			42.2		
Movers core	0.420			0.607		
ROUGE-WE-1	0.403			54.8		
ROUGE-LSUM	0.397			50.8		
ROUGE-WE-2	0.396			40.7		
METEOR	0.388			0.279		
ROUGE-WE-3	0.385			42.5		
RedGraph	0.384			0.397		
PRISM	0.369			−3.24		
ROUGE-3	0.345			20.5		
S^3^-pyr	0.302			0.71		
S^3^-resp	0.301			0.79		
Stats-novel trigram	0.292			0.99		
Stats-density	0.280			6.51		
BLANC	0.165			0.131		
Stats-compression	0.145			8.36		
SUPERT	0.082			0.557		
Stats-coverage	0.078			8.36		
SummaQA	0.075			0.180		

* Lower value represents better performance.
