# Supplementary material for: Generative Large Language Models in Electronic Health Records for Patient Care Since 2023: A Systematic Review
Source: medRxiv. 2024 Aug 19:2024.08.11.24311828. Preprint. [Version 2] doi: 10.1101/2024.08.11.24311828 (PMC11370524; doi:10.1101/2024.08.11.24311828)
Supplement: Supplementary file 1 [file media-1.docx]

**Generative Large Language Models in Electronic Health Records for Patient Care Since 2023: A Systematic Review**

Xinsong Du^1,2,3^, Zhengyang Zhou^4^, Yifei Wang^4^, Ya-Wen Chuang^5,6,7^, Richard Yang^1,2^, Wenyu Zhang^1,2,3^, Xinyi Wang^1,2,3^, Rui Zhang^8^, Pengyu Hong^4^, David W. Bates^1,2,9^, Li Zhou^1,2,3^

^1 ^Division of General Internal Medicine and Primary Care, Brigham and Women's Hospital, Boston, Massachusetts 02115

^2 ^Department of Medicine, Harvard Medical School, Boston, Massachusetts 02115

^3 ^Department of Biomedical Informatics, Harvard Medical School, Boston, Massachusetts 02115

^4 ^Department of Computer Science, Brandeis University, Waltham, MA 02453

^5 ^Division of Nephrology, Department of Internal Medicine, Taichung Veterans General Hospital, Taichung, Taiwan, 407219

^6 ^Department of Post-Baccalaureate Medicine, College of Medicine, National Chung Hsing University, Taichung, Taiwan, 402202

^7 ^School of Medicine, College of Medicine, China Medical University, Taichung, Taiwan, 404328

^8^Division of Computational Health Sciences, University of Minnesota, Minneapolis, MN 55455 

^9^Department of Health Policy and Management, Harvard T.H. Chan School of Public Health, Boston, MA 02115

**Corresponding author:**

Xinsong Du, Ph.D.

Division of General Internal Medicine and Primary Care

Department of Medicine

Brigham and Women’s Hospital and Harvard Medical School

399 Revolution Dr, Suite 777

Somerville, MA 02145

E-mail: xidu1@bwh.harvard.edu

**Supplementary Tables**

| **Table S1.** Search Keywords for Each Database |  |  |
| --- | --- | --- |
| **Database** | **Keywords** | **Search Date** |
| PubMed | ((((((("prompt engineering"[Title/Abstract] OR "large language model"[Title/Abstract] OR "large language models"[Title/Abstract] OR "chatgpt"[Title/Abstract] OR "chatbot"[Title] OR (("machine learning"[Title/Abstract] OR "language"[Title/Abstract] OR "text"[Title/Abstract] OR "artificial intelligence"[Title/Abstract]) AND ("gpt4"[Title/Abstract] OR "gpt-4"[Title/Abstract] OR "llama"[Title/Abstract] OR "claude"[Title/Abstract] OR "bard"[Title/Abstract] OR "falon"[Title/Abstract] OR "cohere"[Title/Abstract] OR "palm"[Title/Abstract]))) NOT "Review"[Publication Type]) NOT "systematic review"[Publication Type]) NOT "meta analysis"[Publication Type]) NOT ("systematic review"[Title/Abstract] OR "meta analysis"[Title/Abstract] OR "Review"[Title/Abstract])) NOT "editorial"[Publication Type]) NOT "comment"[Publication Type]) AND ((fft[Filter]) AND (2023:2024[pdat])) | 05/19/2024 |
| WOS | (TS=("prompt engineering" OR "large language model" OR "large language models" OR "chatgpt" OR (("machine learning" OR "language" OR "text" OR “artificial intelligence”) AND ("gpt4" OR "gpt-4" OR "llama" OR "claude" OR "bard" OR "falon" OR "cohere" OR "palm")))) OR (TI=("chatbot")) AND (PY==("2023" OR "2024")) NOT (DT==("MEETING ABSTRACT" OR “REVIEW ARTICLE” OR “EDITORIAL MATERIAL” OR “CORRECTION” OR “NEWS ITEM” OR “BOOK CHAPTERS” OR “DATA PAPER” OR “REPRINT” OR “BOOK REVIEW” OR “DISCUSSION”)) | 05/19/2024 |

**Supplementary Text – Introduction of Prompt Engineering, Generative LLM Fine-Tuning, and Multimodal Data Integration**

Prompting Methods: Fine-tuning Large Language Models (LLMs) can be very costly ^1^, especially in complex biomedical scenarios.^2^ It's akin to a teacher trying to explain a difficult concept to students. If the teacher only provides a lot of exercises for self-learning, students may struggle, spend a lot of time, and still not grasp the material. In this context, an efficient and low-cost approach to guide the LLM to produce the desired output is essential. Just as a teacher offering explanations, hints, or guiding students through logical steps before they attempt to solve problems can help them understand concepts and solve problems more quickly, prompting methods can similarly be used to redirect and optimize the performance of LLMs. These methods cover a variety of strategies, such as hard-prompt engineering,^3^ soft prompting,^4^ few-shot prompting,^5^ chain-of-thought,^6^ retrieval augmented generation,^7^ and others, demonstrating significant efficacy in enhancing large language models within the biomedical domain.^8^ In our review, we summarized prompting strategies used in included studies.

Fine-Tuning Methods: Fine-tuning large pre-trained language models on downstream tasks has become an important paradigm in NLP. Traditionally, this involves finetuning all parameters in the pre-trained model, which is prohibitively expensive. Parameter-efficient fine-tuning (also called lightweight fine-tuning) has attracted various attention, especially in Transformer-based language models.  Notable fine-tuning methods include adapter tuning^9^ and Low-Rank Adaptation (LoRA).^10^  In particular, LoRA has been widely applied to open-source LLMs as it significantly reduces the number of trainable parameters using low-rank approximation to the update matrix in the dense layer. Many LoRA variants focus on different aspects of improvements, such as improved rank settings and better budget allocation^11^, and more memory-efficient iterations (e.g., QLoRA).^12^ In our review, we summarized fine-tuning strategies used for applying LLM on EHR data.

Multimodal Data Fusion for LLM: Relying solely on textual data for training large language models is inherently restrictive.^13^ Just as doctors diagnosing lung diseases don't rely solely on current clinical notes, but also examine the original chest x-ray images, listen to the patient's description of their symptoms, and review multiple lab test results, incorporating diverse data modalities enables the large language model to comprehend contexts more effectively, akin to having additional senses for problem-solving. Recent research has introduced several effective methods for integrating multimodal data into LLMs, such as CLIP (Contrastive Language-Image Pretraining),^14^ VLP (Visual-Linguistic Pretraining),^15^ UNIMO (Unified Multimodal Pre-training for Language Understanding and Generation),^16^ and Oscar (Object-Semantics Aligned Pre-training).^17^ These approaches enhance the models' ability to understand and generate language in conjunction with diverse data modalities.^18^ Such fusion of multimodal data for LLMs can enhance their performance in complex biomedical scenarios.^19^ In this review, we also summarized papers using LLMs and multimodal EHR data to improve patient care. 

Generative LLM Challenges: LLMs can generate hallucinations, producing content that is inaccurate or inconsistent with real-world facts or user inputs.^20^ Typical detection methods fall into two main categories: factuality hallucination and faithfulness hallucination.^21^ These issues can arise during data preparation, training, and inference. Hallucinations are closely linked to biases in LLMs. For instance, collecting large-scale corpora can inadvertently introduce misinformation and multiple types of biases, such as duplication bias and social bias. Duplication bias could shift LLMs from generation to memorization and would result in hallucinations^22^. Social bias, deriving from negative or toxic views towards certain social groups in internet-based texts, can be an intrinsic factor in hallucinations and may even be amplified by the models. Related studies on the subcategories of social bias in language models include gender bias,^23^ nationality bias,^24^ racial bias,^25^ and bias against stigmatized groups.^26^

**References**

1.	Ding N, Qin Y, Yang G, et al. Parameter-efficient fine-tuning of large-scale pre-trained language models. *Nat Mach Intell*. 2023;5(3):220-235. doi:10.1038/s42256-023-00626-4

2.	Thirunavukarasu AJ, Ting DSJ, Elangovan K, Gutierrez L, Tan TF, Ting DSW. Large language models in medicine. *Nat Med*. 2023;29(8):1930-1940. doi:10.1038/s41591-023-02448-8

3.	Wen Y, Jain N, Kirchenbauer J, Goldblum M, Geiping J, Goldstein T. Hard Prompts Made Easy: Gradient-Based Discrete Optimization for Prompt Tuning and Discovery. *Adv Neural Inf Process Syst*. 2023;36:51008-51025.

4.	Yang JC, Li Z, Xie S, Yu W, Li S, Du B. Soft-Prompting with Graph-of-Thought for Multi-modal Representation Learning. In: Calzolari N, Kan MY, Hoste V, Lenci A, Sakti S, Xue N, eds. *Proceedings of the 2024 Joint International Conference on Computational Linguistics, Language Resources and Evaluation (LREC-COLING 2024)*. ELRA and ICCL; 2024:15024-15036. Accessed July 25, 2024. https://aclanthology.org/2024.lrec-main.1306

5.	Nori H, Lee YT, Zhang S, et al. Can Generalist Foundation Models Outcompete Special-Purpose Tuning? Case Study in Medicine. Published online November 27, 2023. doi:10.48550/arXiv.2311.16452

6.	Liu P, Yuan W, Fu J, Jiang Z, Hayashi H, Neubig G. Pre-train, Prompt, and Predict: A Systematic Survey of Prompting Methods in Natural Language Processing. *ACM Comput Surv*. 2023;55(9):195:1-195:35. doi:10.1145/3560815

7.	Unlu O, Shin J, Mailly CJ, et al. Retrieval-Augmented Generation–Enabled GPT-4 for Clinical Trial Screening. *NEJM AI*. 2024;1(7):AIoa2400181. doi:10.1056/AIoa2400181

8.	Hu Y, Chen Q, Du J, et al. Improving large language models for clinical named entity recognition via prompt engineering. *J Am Med Inform Assoc*. Published online January 27, 2024:ocad259. doi:10.1093/jamia/ocad259

9.	Houlsby N, Giurgiu A, Jastrzebski S, et al. Parameter-Efficient Transfer Learning for NLP. Published online June 13, 2019. doi:10.48550/arXiv.1902.00751

10.	Hu EJ, Shen Y, Wallis P, et al. LoRA: Low-Rank Adaptation of Large Language Models. Published online October 16, 2021. doi:10.48550/arXiv.2106.09685

11.	Zhang Q, Chen M, Bukharin A, et al. AdaLoRA: Adaptive Budget Allocation for Parameter-Efficient Fine-Tuning. Published online December 20, 2023. doi:10.48550/arXiv.2303.10512

12.	Dettmers T, Pagnoni A, Holtzman A, Zettlemoyer L. QLoRA: Efficient Finetuning of Quantized LLMs. *Adv Neural Inf Process Syst*. 2023;36:10088-10115.

13.	Wu J, Gan W, Chen Z, Wan S, Yu PS. Multimodal Large Language Models: A Survey. Accessed July 25, 2024. https://www.computer.org/csdl/proceedings-article/bigdata/2023/10386743/1TUOsrxd62A

14.	Radford A, Kim JW, Hallacy C, et al. Learning Transferable Visual Models From Natural Language Supervision. *arXiv.org*. Published online February 26, 2021. Accessed July 25, 2024. https://www-proquest-com.ezp-prod1.hul.harvard.edu/docview/2495203947?parentSessionId=3Z%2B%2BA67quBDJ6Adc8hcWQ5PuO6yoDyLwXCjyEDPMBy4%3D&pq-origsite=primo&sourcetype=Working%20Papers

15.	Zhou L, Palangi H, Zhang L, Hu H, Corso JJ, Gao J. Unified Vision-Language Pre-Training for Image Captioning and VQA. arXiv.org. September 24, 2019. Accessed July 25, 2024. https://arxiv.org/abs/1909.11059v3

16.	Li W, Gao C, Niu G, et al. UNIMO: Towards Unified-Modal Understanding and Generation via Cross-Modal Contrastive Learning. In: Zong C, Xia F, Li W, Navigli R, eds. *Proceedings of the 59th Annual Meeting of the Association for Computational Linguistics and the 11th International Joint Conference on Natural Language Processing (Volume 1: Long Papers)*. Association for Computational Linguistics; 2021:2592-2607. doi:10.18653/v1/2021.acl-long.202

17.	Li X, Yin X, Li C, et al. Oscar: Object-Semantics Aligned Pre-training for Vision-Language Tasks. arXiv.org. April 13, 2020. Accessed July 25, 2024. https://arxiv.org/abs/2004.06165v5

18.	Acosta JN, Falcone GJ, Rajpurkar P, Topol EJ. Multimodal biomedical AI. *Nat Med*. 2022;28(9):1773-1784. doi:10.1038/s41591-022-01981-2

19.	Rieffel J, Chitgupi U, Lovell JF. Recent Advances in Higher-Order, Multimodal, Biomedical Imaging Agents. *Small*. 2015;11(35):4445-4461. doi:10.1002/smll.201500735

20.	Bang Y, Cahyawijaya S, Lee N, et al. A Multitask, Multilingual, Multimodal Evaluation of ChatGPT on Reasoning, Hallucination, and Interactivity. In: Park JC, Arase Y, Hu B, et al., eds. *Proceedings of the 13th International Joint Conference on Natural Language Processing and the 3rd Conference of the Asia-Pacific Chapter of the Association for Computational Linguistics (Volume 1: Long Papers)*. Association for Computational Linguistics; 2023:675-718. doi:10.18653/v1/2023.ijcnlp-main.45

21.	Huang L, Yu W, Ma W, et al. A Survey on Hallucination in Large Language Models: Principles, Taxonomy, Challenges, and Open Questions. Published online November 9, 2023. doi:10.48550/arXiv.2311.05232

22.	Hernandez D, Brown T, Conerly T, et al. Scaling Laws and Interpretability of Learning from Repeated Data. Published online May 20, 2022. doi:10.48550/arXiv.2205.10487

23.	Paullada A, Raji ID, Bender EM, Denton E, Hanna A. Data and its (dis)contents: A survey of dataset development and use in machine learning research. *Patterns*. 2021;2(11):100336. doi:10.1016/j.patter.2021.100336

24.	Venkit PN, Gautam S, Panchanadikar R, Huang TH “Kenneth”, Wilson S. Nationality Bias in Text Generation. Published online February 14, 2023. doi:10.48550/arXiv.2302.02463

25.	Blodgett SL, O’Connor B. Racial Disparity in Natural Language Processing: A Case Study of Social Media African-American English. Published online June 30, 2017. doi:10.48550/arXiv.1707.00061

26.	Mei K, Fereidooni S, Caliskan A. Bias Against 93 Stigmatized Groups in Masked Language Models and Downstream Sentiment Classification Tasks. In: *2023 ACM Conference on Fairness, Accountability, and Transparency*. ACM; 2023:1699-1710. doi:10.1145/3593013.3594109
